# Immunological evaluation of vestibular folds in autopsies of patients with the Acquired Immunodeficiency Syndrome

**DOI:** 10.1016/S1808-8694(15)30569-3

**Published:** 2015-10-19

**Authors:** Janaínna Grazielle Pacheco Olegário, Renata Calciolari Rossi, Ana Karina Marques Salge, Rosana Rosa Miranda Corrêa, Vicente de Paula Antunes Teixeira, Eumenia Costa da Cunha Castro

**Affiliations:** 1 Graduated in biomedicine, master's degree student.; 2 Master's degree in general pathology, doctoral student.; 3 Doctor in general pathology, adjunct professor of the nursing department, Goiaas Federal University (Universidade Federal de Goiás).; 4 Doctor in general pathology, adjunct professor of general pathology, Triangulo Mineiro Federal University (Universidade Federal do Triângulo Mineiro).; 5 Doctor, full professor of general pathology, Triangulo Mineiro Federal University (Universidade Federal do Triângulo Mineiro).; 6 Post-doctor, adjunct professor of general pathology Triangulo Mineiro Federal University (Universidade Federal do Triângulo Mineiro).

**Keywords:** autopsy, vestibular folds, aids

## Abstract

Immune response cells are decreased in patients with the Acquired Immunodeficiency Syndrome. This alters the cell population in vestibular fold lymphoid follicles, leading to respiratory infections in these patients. Such infections are the main cause of mortality and morbidity in these patients.

**Aim:**

to characterize lymphoid follicle cell populations in the vestibular folds of adults with the Acquired Immunodeficiency Syndrome and associated or not respiratory infection.

**Materials and methods:**

A retrospective study was made of 64 adult larynges harvested during routine autopsies. Anti-B cell, Anti-CD3, Anti-CD68 and Anti-follicular dendritic cell antibodies were used for immunological testing.

**Results:**

46 (71.87%) of the sample patients had the Acquired Immunodeficiency Syndrome. In these patients, lymphoid follicle cellularity was lower compared to the control group. The cell number was decreased in patients with the Acquired Immunodefficiency Syndrome and associated respiratory tract infection.

**Conclusion:**

We demonstrated in this study that vestibular fold lymphoid follicles were affected by viral infections, and may be considered as a reliable marker of immunodepression in these patients.

## INTRODUCTION

The laryngeal cavity is divided into three compartments: the supraglottis, the glottis and the subglottis. The vestibular folds (VF) are found in the supraglottic area.^1^, ^2^, ^3^ The VF are two sagittally oriented thick laminae with a double-sized mucosa, which arises within the supraglottic wall.^1^ Their purpose is to lubricate and isolate the true vocal folds, making it possible for them to vibrate. It is thought that VF - due to its lymphoid follicles (LF) - also act as a barrier preventing infectious agents from reaching the lower airways.^2^, ^3^

The role of LF found close to mucosae is to produce substances that activate an immune response against infectious agents in the mucosa.^4^, ^5^, ^6^ Cytokines produced in LF promote plasmacytic differentiation and antibody production - mostly IgA in the respiratory tract - that are excreted in mucosal cell secretions,^4^, ^5^, ^6^ including the VF mucosa.^7^

According to the literature, the role of LF in VF in immunocompetent patients is to protect upper airways, similar to mucosa-associated lymphoid tissue.^2^, ^7^, ^8^, ^9^, ^10^

There are various changes in HIV-infected individuals, which have fewer and dysfunctional CD4 T lymphocytes. There have been reports suggesting that the remaining immune cells in HIV-immunodepressed individuals act as viral reservoirs; in this situation, lymphoid tissues, including those in the larynx, would be viral replication structures.^11^, ^12^ Other studies have reported that CD8 T lymphocytes are more numerous in the same context, although the total number of immune cells is decreased.^9^ We found no reports in the literature, however, about the cell phenotype that forms the lymphoid organs located in the VF.

It is known that lymphoid tissue associated with IgA-secreting mucosa is the main local protection mechanism in the respiratory tract, and that vaccines aiming to provide upper airway immunity against respiratory infection seek to activate this mechanism.^4^, ^5^, ^6^

Respiratory infections are the main cause of mortality and morbidity in HIV patients.^13^, ^14^ Understanding the nature of cells in LF within VF is the first step to use the immune system located in mucosae as protection against respiratory infections in AIDS patients.

The purpose of this study was to characterize the cell population in LF located in the VF of autopsied AIDS adults with and with no associated respiratory infection.

## MATERIAL AND METHOD

A retrospective cross-sectional study was made of 285 adult autopsies done between 1993 and 2003. The patients were divided into two groups. The first group included all of the AIDS patients whose larynges had been harvested during the autopsy; complete files and autopsy reports were also included. This group was composed of 46 cases. The control group included AIDS-free patients paired by age with the first group; cases where files or autopsy reports were incomplete and those from which larynges were not harvested were excluded. The control group was composed of 18 cases. The files provided information about underlying diseases, gender and age; autopsy reports elucidated the causes of death. The 64 autopsies were subdivided, according to the cause of death, into patients with and patients with no respiratory infection. The Research Ethics Committee approved this study on 25/06/2004, protocol number 481.

Laringes were harvested during routine autopsies and fixed in 4% formaldehyde, after which they were cut above and below the glottic cavity; the distance between cuts was 3 cm. This fragment, which contained the VF, was sectioned frontally in parallel and processed for paraffin inclusion.

B and T lymphocyte, macrophage and follicular dendritic cell immunohistochemistry was used for the morphometric analysis, using the KS300 software (KONRONZEISS, Germany) for quantification. Immunohistochemical methods used the primary Anti-B-Cells (Biogenex, EUA), Anti-CD3 (Dako, EUA), Anti-CD68 (Dako, EUA) and Anti-Follicular Dendritic Cell (Dako, EUA) antibodies. Slides were deparaffinized in xylol and hydrated in descending concentrations of alcohol. Antigen recovery was done with a citrate buffer at 97°C for Anti-B-Cells, Anti-CD68 and Anti-Follicular Dendritic Cell antibodies, and Trypsin at room temperature for the Anti-CD3 antibody. The PBS 0,05M + Triton X-100 0,05% buffer was used for slide lavage. All antibodies were incubated during a mean 12 hours; the secondary antibody was incubated for about 2 hours. The Avidina-Biotina complex (Dako, EUA) remained in contact with the sections during about 30 minutes. The material was incubated in 3’3 Diaminobenzidine (DAB) for processing, after which it was Hematoxyllin-stained and mounted in synthetic entelan media.

The Sigmastat software was used for the statistical analysis. In the comparison between two groups, Student's “t” test was used for normal variables and the Mann-Whitney test was used for non-normal variables. Statistically significant differences were those in which the probability of rejection of the null hypothesis was below 5% (0.05).

## RESULTS

There were 64 VF of autopsied adults. Of these, 46 cases (71.87%) were diagnosed with AIDS. Within this group, the cause of death was upper airway infection in 33 cases (51.6%). The control group consisted of 18 cases (28.1%), of which 10 cases (15.6%) died due to upper airway infection.

The group of AIDS patients had less B lymphocytes (p=0.292) (Figures 1 and 2), T lymphocytes (p=0.007) (Figures 3 and 4), macrophages (p=0.033) (Figures 5 and 6) and dendritic cells (p=0.048) (Figures 7 and 8) (Table 1) compared to the control group.

**Figure 1:**
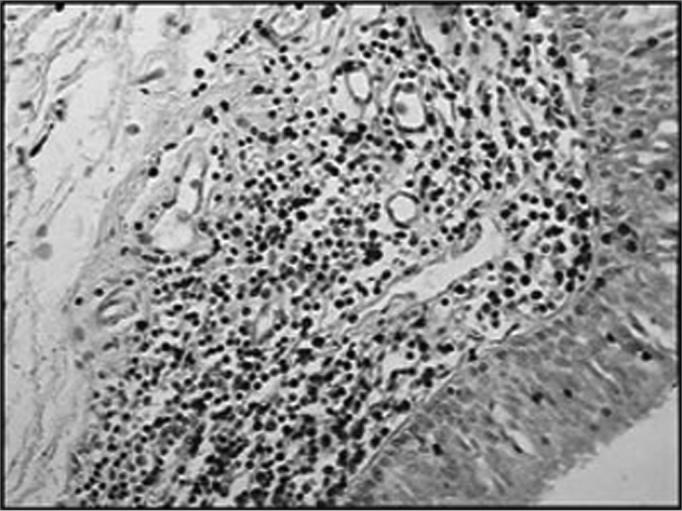
Immunohistochemistry for B lymphocytes in vestibular folds - AIDS-free patients with peritonitis as the cause of death (PAP X200)

**Figure 2:**
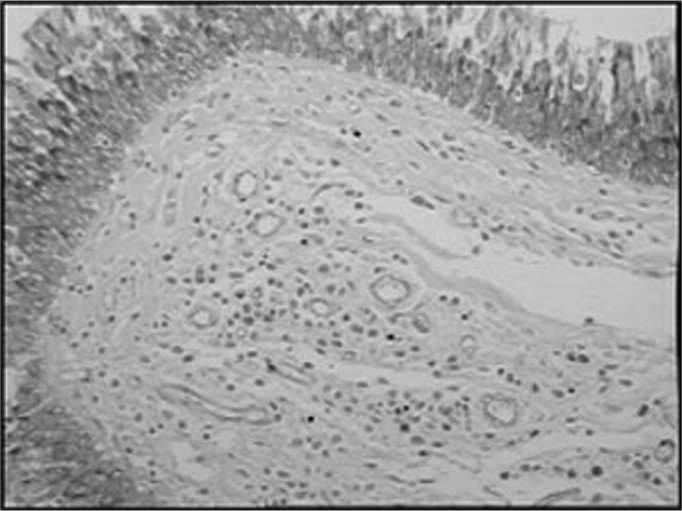
Immunohistochemistry for B lymphocytes in vestibular folds - AIDS patients with endocarditis as the cause of death (PAPX200)

**Figure 3:**
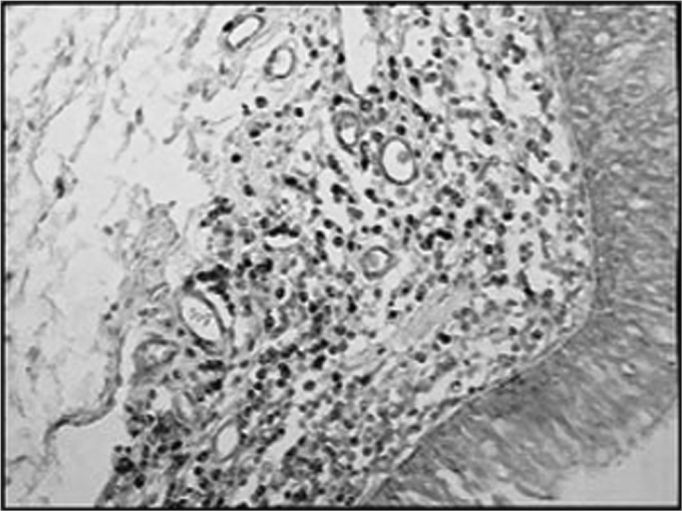
Immunohistochemistry for T lymphocytes in vestibular folds - AIDS-free patients with peritonitis as the cause of death (PAP X200)

**Figure 4:**
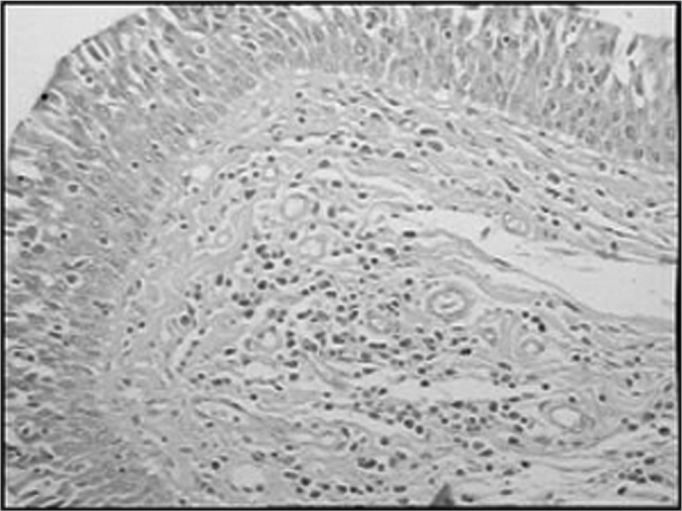
Immunohistochemistry for T lymphocytes in vestibular folds - AIDS patients with endocarditis as the cause of death (PAP X200)

**Figure 5:**
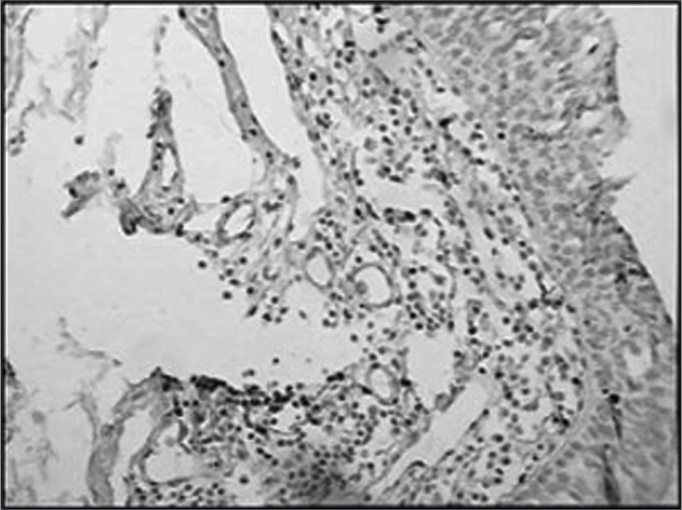
Immunohistochemistry for macrophages (CD68) in vestibular folds - AIDS-free patients with peritonitis as the cause of death (PAP X200)

**Figure 6:**
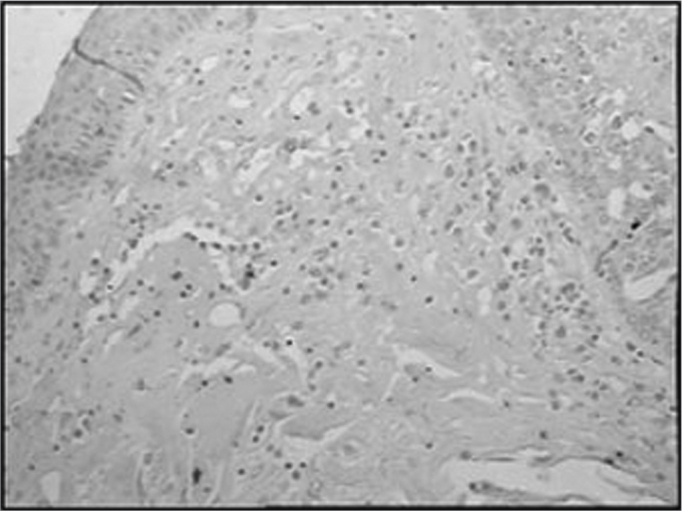
Immunohistochemistry for macrophages (CD68) in vestibular folds - AIDS patients with endocarditis as the cause of death (PAP X200)

**Figure 7:**
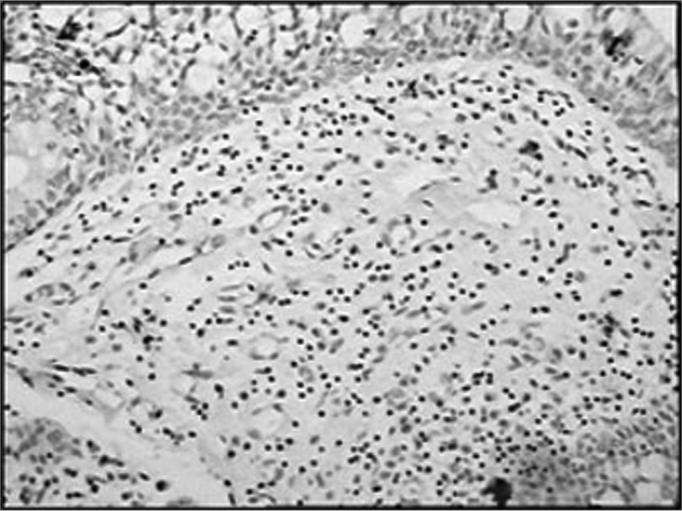
Immunohistochemistry for follicular dendritic cells (anti-FDC) in vestibular folds - AIDS-free patients with peritonitis as the cause of death (PAP X200)

**Figure 8:**
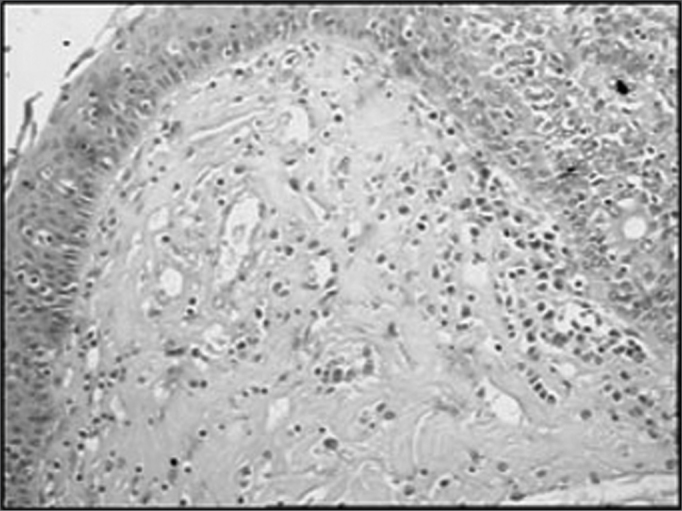
Immunohistochemistry for follicular dendritic cells (anti-FDC) in vestibular folds - AIDS patients with endocarditis as the cause of death (PAP X200)

**Table 1:** Comparison between cell number and the presence or absence of AIDS in autopsied patients between 1993 and 2003.

Number of cells		
	Med (Vmin-Vmax)	
	AIDS n(%) No AIDS n(%)	
	46(71,9) 18(28,1)	
B lymphocytes^A^ (10^3^)	5,2(1,9-11,4)	8,3(3,5-13,4)
T lymphocytes^B^ (10^3^)	3,6 (1,4-7,9)	8,7 (3,6-15,6)
Macrophages^C^	51,5 (19,0-110,0)	81,0 (50,0-352,0)
Dendritic cells^D^	0,0 (0,0-1,0)	1,0 (0,0-2,0)

AT= 656.000; p= 0.292. BT 765.000; p= 0.007; CT= 728.500; p= 0.033. DT= 717.500; p= 0.048.

AIDS= Acquired Immunodefficiency Syndrome; n= number of cases; Med= median; Vmin= minimum value; Vmax= maximum value; (103)= Values raised to the power of three.

The number of mononuclear cells was lower in AIDS patients that had respiratory infection compared to AIDS-free patients with respiratory infection. This difference was statistically significant in the case of T lymphocytes (p=0.024) (Table 2).

**Table 2:** Comparison between cell number and the diagnosis of AIDS in patients with respiratory infections autopsied between 1993 and 2003.

Respiratory infection		
	Med (Vmin-Vmax)	
	AIDS n(%)	No AIDS n(%)
	33 (51,6)	10 (15,6)
B lymphocytes^A^ (10^3^)	4,9 (2,1-10,7)	12,7 (5,9-16,5)
T lymphocytes^B^ (10^3^)	2,6 (1,1-5,4)	6,9 (3,4-9,9)
Macrophages^C^	49,0 (16,3-111,0)	71,5 (43,0-352,0)
Dendritic cells^D^	0,0 (0,0-0,0)	1,0 (0,0-1,0)

AT= 271.000; p= 0.147. BT= 299.000; p= 0.024. CT= 276.000; p= 0.237. DT = 261.500; p = 0.237.

AIDS= Acquired Immunodefficiency Syndrome; n= number of cases; Med= median; Vmin= minimum value; Vmax= maximum value; (103)= Values raised to the power of three.

AIDS patients with respiratory infection had fewer of all cell types compared to AIDS patients with no respiratory infection; this difference was statistically significant in relation to T lymphocytes (p=0.019) (Table 3).

**Table 3:** Comparison between cell number and the diagnosis of respiratory infection in AIDS patients autopsied between 1993 and 2003.

AIDS		
	Com infecção respiratória n(%)	
	Sem infecção respiratória n(%)	
	33 (51,6)	13 (20,3)
B lymphocytes^A^ (10^3^)	4,9 (2,1-10,7)	5,7 (1,8-14,7)
T lymphocytes^B^ (10^3^)	2,6 (1,1-5,4)	5,9 (3,8-12,7)
Macrophages^C^	68,3±65,8	79,4±60,3
Dendritic cells^D^	0,0 (0,0-1,0)	0,0 (0,0-1,0)

AT= 296.000; p= 0.826. BT= 402.000; p= 0.019. Ct= 0.524; p= 0.603. DT= 295.000; p= 0.807. AIDS= Acquired Immunodefficiency Syndrome; n= number of cases; (103)= Values raised to the power of three.

## DISCUSSION

AIDS features progressive CD4 T lymphocyte depletion and dysfunction, with a resulting decline in immunocompetency.^12^, ^15^ This study aimed to immunohistochemically describe the population of mononuclear cells in adult autopsied LF of VF, with AIDS as the underlying disease.

We found that in AIDS patients, macrophages, follicular dendritic cells, and T and B lymphocytes are fewer in the LF of VF compared with AIDS-free patients. AIDS virus tropism is for T lymphocytes, macrophages, Langherans cells, follicular dendritic cells and endothelial cells located in lymphoid organs. Depletion of these cells increases with the severity of virus infection. Studies of lymphoid organs in AIDS patients at advanced stages of this disease have shown that LF and tonsils had marked lymphocytic depletion and weakly defined or non-existent germinative centers, which is in agreement with our findings in this study. Various authors have suggested that germinative centers may be essential structures for viral replication. Destruction of follicular dendritic cells is a constant immunoarchitectural finding in AIDS-related lymphadenopathy.^11^, ^12^ This was also found in the LF of VF in this study.

In immunocompetent patients, lung infection is associated with increased T and B cells in LF of VF.^16^ In the current study, AIDS patients with respiratory infections had fewer mononucleated cells as a whole compared with AIDS-free patients and AIDS-free patients with no respiratory infection. Lymphocytes and mononucleated cells are the main reservoirs of the HIV. These cells act as reservoirs that favor virus persistence and transmission.^11^, ^12^, ^17^ Germinative centers disappear with progressive immune suppression, which increases immunodepression and at the same time increasing the number of circulating viruses. This situation increases the severity of infection in other organs in advanced stages of the disease.^9^ In patients with respiratory infections, a decreased number of inflammatory cells in LF of VF indicates that associated infection occurs in patients with more severe immunodepression, which already had fewer cells in LF of VF to begin with.

## CONCLUSION

This study demonstrated that LF of VF are affected by virus infection, and that they reliably represent the immunological status of these patients.
